# Adipose tissue depot specific expression and regulation of fibrosis-related genes and proteins in experimental obesity

**DOI:** 10.1007/s00335-023-10022-3

**Published:** 2023-10-26

**Authors:** Kristina Eisinger, Philipp Girke, Christa Buechler, Sabrina Krautbauer

**Affiliations:** 1 Department of Internal Medicine I, Regensburg University Hospital, 93053 Regensburg, Germany; 2 Department of Genetics, University of Regensburg, 93040 Regensburg, Germany

**Keywords:** Bambi, Actin alpha 2 smooth muscle, Collagen, White fat, Brown fat, Lipopolysaccharide

## Abstract

Transforming growth factor beta (Tgfb) is a well-studied pro-fibrotic cytokine, which upregulates *cellular communication network factor 2* (*Ccn2*), *collagen*, and *actin alpha 2, smooth muscle* (*Acta2*) expression. Obesity induces adipose tissue fibrosis, which contributes to metabolic diseases. This work aimed to analyze the expression of *Tgfb*, *Ccn2*, *collagen1a1* (*Col1a1*), *Acta2* and *BMP and activin membrane-bound inhibitor* (*Bambi*), which is a negative regulator of Tgfb signaling, in different adipose tissue depots of mice fed a standard chow, mice fed a high fat diet (HFD) and ob/ob mice. Principally, these genes were low expressed in brown adipose tissues and this difference was less evident for the ob/ob mice. Ccn2 and Bambi protein as well as mRNA expression, and *collagen1a1* mRNA were not induced in the adipose tissues upon HFD feeding whereas *Tgfb* and *Acta2* mRNA increased in the white fat depots. Immunoblot analysis showed that Acta2 protein was higher in subcutaneous and perirenal fat of these mice. In the ob/ob mice, *Ccn2* mRNA and Ccn2 protein were upregulated in the fat depots. Here, *Tgfb*, *Acta2* and *Col1a1* mRNA levels and serum Tgfb protein were increased. Acta2 protein was, however, not higher in subcutaneous and perirenal fat of these mice. *Col6a1* mRNA was shown before to be higher in obese fat tissues. Current analysis proved the Col6a1 protein was induced in subcutaneous fat of HFD fed mice. Notably, Col6a1 was reduced in perirenal fat of ob/ob mice in comparison to the respective controls. 3T3-L1 cells express Ccn2 and Bambi protein, whose levels were not changed by fatty acids, leptin, lipopolysaccharide, tumor necrosis factor and interleukin-6. All of these factors led to higher Tgfb in 3T3-L1 adipocyte media but did not increase its mRNA levels. Free fatty acids induced necrosis whereas apoptosis did not occur in any of the in vitro incubations excluding cell death as a main reason for higher Tgfb in cell media. In summary, *Tgfb* mRNA is consistently induced in white fat tissues in obesity but this is not paralleled by a clear increase of its target genes. Moreover, discrepancies between mRNA and protein expression of Acta2 were observed. Adipocytes seemingly do not contribute to higher *Tgfb* mRNA levels in obesity. These cells release more Tgfb protein when challenged with obesity-related metabolites connecting metabolic dysfunction and fibrosis.

**Supplementary Information:**

The online version contains supplementary material available at 10.1007/s00335-023-10022-3.

## Introduction

In obesity, storage of surplus fat in adipose tissues is managed by the growth of resident adipocytes and the increase in adipocyte number. Massive expansion of adipose tissues is associated with immune cell infiltration and the remodeling of extracellular matrix. Deposition of excess extracellular matrix proteins limits the capacity of adipocytes to expand, and fatty acids have to be stored in other organs such as the liver and skeletal muscle (Buechler et al. 2015; Sun et al. 2013). Lipid storage in peripheral organs is a main cause of insulin resistance and metabolic diseases (Buechler et al. 2015; Sun et al. 2013).

Transforming growth factor beta (Tgfb) is an extensively studied fibrotic cytokine. Tgfb induces myofibrogenesis, and myofibroblasts express actin alpha 2, smooth muscle (Acta2, also named alpha-smooth muscle actin) and produce extracellular matrix proteins such as collagens (Lee 2018; Marcelin et al. 2019). In adipose tissues, adipocyte progenitor cells develop a myofibroblast phenotype upon Tgfb stimulation (Lee 2018; Lin et al. 2018). These cells express Acta2, which is upregulated by Tgfb (Lee et al. 2006). Tgfb, moreover, inhibits adipocyte differentiation and increases cell growth (Choy et al. 2000). Notably, blockage of Tgfb signaling protected from weight gain and metabolic disease in an experimental model (Yadav et al. 2011). Adipocytes and stromal vascular cells such as macrophages produce Tgfb and its adipose tissue expression increases in obesity (Choy et al. 2000; Yadav et al. 2011; Fain et al. 2005). Hence, higher serum TGFb in obese humans may originate from adipose tissues (Lee 2018). It is important to note that TGFb is an anti-inflammatory cytokine and may also protect from fibrosis by lowering tissue inflammation. TGFb, in addition, inhibits cell proliferation, which is essential for regenerative processes. TGFb activation and signaling are tightly regulated and higher expression of *Tgfb* does not prove increased biologic activity (Weber et al. 2023).

Tgfb is a transcriptional regulator of *cellular communication network factor 2* (*Ccn2*), and Tgfb as well as Ccn2 inhibit adipogenesis (Choy et al. 2000; Tan et al. 2008; Gressner et al. 2008; Ignotz and Massague 1985). Consequently, *Ccn2* expression is downregulated in mature adipocytes in comparison to preadipocytes (Tan et al. 2008). The stromal vascular fraction containing progenitor cells, preadipocytes, endothelial cells and immune cells, expressed about fivefold more *CCN2* than human adipocytes (Yoshino et al. 2019; Buechler and Schaffler 2007). *Ccn2*/CCN2 expression was induced in adipose tissues of obese rodents and humans, and CCN2 protein was found associated with adipose tissue fibrosis in human obesity (Yoshino et al. 2019; Tan et al. 2013).

In mice, *Ccn2* was about twofold higher expressed in epididymal than subcutaneous fat (Tan et al. 2008). Intra-abdominal fat depots such as epididymal and perirenal fat differ from each other and from subcutaneous fat. Excess intra-abdominal fat is related to insulin resistance and metabolic diseases whereas subcutaneous fat is rather protective (Mardian et al. 2017; Sierra Rojas et al. 2016; Tran et al. 2008). The physiological function of elevated *Ccn2* expression in epididymal fat needs further study (Tan et al. 2008; Ibrahim 2010).

BMP and activin membrane-bound inhibitor (Bambi) is a pseudoreceptor of the Tgfb type I receptor and functions as a negative regulator of Tgfb signalling (Sekiya et al. 2004). *Bambi* mRNA is expressed in the stromal vascular cell fraction and in adipocytes of pigs, and is about three times less abundant in these latter cells (Mai et al. 2014). Tgfb inhibits adipogenesis (Choy et al. 2000) suggesting that impaired Tgfb signalling by Bambi may promote adipocyte differentiation. Unexpectedly, Bambi blockage, and not overexpression, had this effect in *in vitro* models, and *Bambi* mRNA as well as Bambi protein levels thus declined during adipocyte differentiation (Mai et al. 2014; Luo et al. 2012; Yang et al. 2020).

Adipocyte-specific Bambi knock-out mice fed a high fat diet were more obese, and suffered from metabolic disease. Notably, adipocyte area of white and brown fat was greatly increased and both fat tissues accumulated more lipids compared to controls (Chen et al. 2021). Low *Bambi* expression in epididymal fat of diet-induced obese and ob/ob mice thus may promote adipogenesis and adipose tissue growth (Luo et al. 2012).

Collagens are main components of the extracellular matrix, which can accumulate around the fat cells or form collagen fibers (Marcelin et al. 2019). Collagen (Col) I, IV and VI are the dominantly expressed collagens in murine fat tissues (Huber et al. 2007). Experiments using *Col6* deficient ob/ob mice suggest that accumulation of Col6 limits the expansion of fat cells (Khan et al. 2009). In the mutant mice, uninhibited growth of adipocytes was associated with improved metabolic health (Khan et al. 2009). It was also described that mice with knock-down of *collagen type VI, alpha3* (*Col6a3*) had less epididymal fat mass and small adipocytes in accordance with a function of Col6a3 in adipogenesis and lipolysis (Oh et al. 2021). *Col1a1*, *Col1a2*, *Col4a1*, *Col5a1*, *Col5a2*, *Col5a3*, *Col6a1* and *Col6a3* mRNA levels were found to be at least 1.3-fold induced in adipose tissues of db/db mice, which are obese because of dysfunctional leptin signalling, in comparison to wild type animals (Khan et al. 2009). *Col1a2*, *Col3a1*, *Col5a2*, *Col6a3* and *Col8a1* were higher expressed in adipose tissues of db/db mice fed a high fat diet (Huber et al. 2007). Collagen deposition has been also linked with adipose tissue dysfunction in humans (Buechler et al. 2015; Marcelin et al. 2019). Notably, fibrosis in subcutaneous fat was associated with resistance to bariatric surgery related weight loss (Divoux et al. 2010). *Col6a3* mRNA expression was, however, reduced in obese subcutaneous and omental adipose tissues (McCulloch et al. 2015).

Mouse strains differ in the susceptibility to develop adipose tissue fibrosis. Expression of *Col1a1* and *Col3a1* was higher in epididymal fat of C3H/He mice than of C57BL/6 mice when both strains were fed a high fat diet. *Col6a1* was, however, specifically increased in epididymal adipose tissue of the C57BL/6 mice fed a high fat diet (Marcelin et al. 2017). The C57BL/6 strain is widely used (Bryant 2011) and regulation of fibrotic genes in fat tissues of obese mice has been also described in this strain (Marcelin et al. 2017; Martinez-Huenchullan et al. 2019). Leptin-deficient ob/ob mice are often maintained on a C57BL/6 genetic background (Ewart-Toland et al. 1999), and for comparison animals with the identical background have to be analyzed. Although C57BL/6 mice are less prone to develop adipose tissue fibrosis (Marcelin et al. 2017), this strain is commonly used in obesity research (Moura et al. 2021) and adipose tissue dysfunction and higher expression of fibrotic genes in obesity has been shown before (Marcelin et al. 2017; Song et al. 2023).

White adipose tissue is organized into different depots, which vary in cellular composition and function. Brown adipose tissue is quite different from white fat and helps to maintain body temperature by burning fatty acids (Lo and Sun 2013; Sanchez-Gurmaches and Guertin 2014). In this study, co-regulation of the genes described above was analyzed in murine subcutaneous, intra-abdominal and brown adipose tissues. Mice fed a normal chow, mice fed a high fat diet and extremely obese ob/ob mice were studied. In 3T3-L1 adipocytes, the effects of obesity-related factors (free fatty acids, leptin, and inflammatory molecules) on the mRNA and protein levels of Tgfb, Ccn2 and Bambi were analyzed.

## Materials and methods

### Murine adipose tissue

Mice were ordered from Charles River Laboratories (Sulzfeld, Germany) and housed with 3–5 mice per cage in a 21 ± 1 °C controlled room under a 12 h light–dark cycle. Animals had free access to food and water. Rising concentrations of CO_2_ produced loss of consciousness and was followed by cervical dislocation.

C57BL/6NCrl mice at the age of 7 weeks were subsequently fed either a high fat diet (HFD, 7 mice) or standard chow (SD, 7 mice) for 14 weeks. Tissues of these animals were used for immunoblot experiments and the purified total RNA was used for analysis of gene expression by real-time RT-PCR. C57BL/6 NCrl mice at the age of 14 weeks were subsequently fed a HFD or SD for 14 weeks, and subcutaneous fat of these mice was used to isolate RNA for GeneChip analysis (Eisinger et al. 2018). Final body weight of the 5 mice on HFD was 39.3 (32.5–41.3) g and of the 5 mice on SD was 25.6 (23.9–27.1) g (*p* = 0.009). Leptin-deficient male ob/ob mice (JAX^®^ Mice Strain) on the C57BL/6 background (5 animals) and the respective wild type mice (WT, 5 animals) were obtained at an age of 10 weeks, and were killed 3 weeks later. Mice were killed after overnight fasting and fat tissues were immediately removed and stored at − 80 °C.

### Reagents

Lipopolysaccharide (LPS) (*Escherichia coli* serotype 055:B5), palmitic acid (PA) and oleic acid (OA) were ordered from Sigma (Deisenhofen, Germany). Recombinant tumor necrosis factor (TNF), interleukin (IL)-6 and leptin, and mouse Tgfb DuoSet ELISA were from R&D Systems (Wiesbaden-Nordenstadt, Germany). Ccn2 ELISA was from Hölzel Diagnostika Handels GmbH (Köln, Germany).

### Adipocyte cell culture

The 3T3-L1 preadipocytes were ordered from the American Type Culture Collection (ATCC, Manassas, VA, USA). The cells were cultivated at 37 °C and 5% CO_2_ in Dulbecco’s Modified Eagle Medium (Biochrom, Berlin, Germany) supplemented with 10% newborn calf serum (Sigma Bioscience, Deisenhofen, Germany) and 1% penicillin/streptomycin (PAN, Aidenbach, Germany). For adipogenesis, 3T3-L1 preadipocytes were grown to confluence and differentiated into adipocytes as described (Bauer et al. 2011). To study the effect of inflammatory factors during differentiation, the substances were added at day 0 (start of differentiation) and medium was changed at day 3, 6, 7 and 8 during differentiation.

### Fatty acid treatment

A fatty acid stock solution (200 mM) was prepared in ethanol by heating at 70 °C and 100 μl was added to 900 μl of a 10% fatty acid-free bovine serum albumin solution (BSA, Roche, Mannheim, Germany) to obtain a 20 mM stock solution. The BSA-bound fatty acid stock solution or equal amounts of BSA were added to the differentiated 3T3-L1 adipocytes for 24 h. To study the effect of fatty acids during differentiation, the fatty acid solution was added at day 0 (start of differentiation) and medium was changed at day 3, 6, 7 and 8 during differentiation.

### SDS-PAGE and immunoblotting

Adipose tissues were solubilized in radioimmunoprecipitation assay lysis buffer (50 mM Tris HCl, pH 7.4, 150 mM NaCl, 5 mM EDTA, 0.05% v/v Nonidet P-40, 1% v/v sodium deoxycholate, 1% v/v Triton X-100 and 0.1% v/v SDS). Twenty µg protein was separated by SDS-PAGE and was transferred to PVDF membranes (Bio-Rad, Germany). Incubations with antibodies were performed in 1% BSA in PBS, 0.1% Tween overnight. Immunodetection was done by the ECL Western blot detection system (Amersham Pharmacia, Deisenhofen, Germany). Antibodies to GAPDH, poly(ADP-ribose) polymerase-1 (PARP), collagen 1a1 and cyclophilin A were from New England Biolabs GmbH (Frankfurt, Germany). Ccn2 antibody was from Novus Biologicals (Cambridge, UK) and Bambi antibody from Abcam (Cambridge, UK). Collagen VI alpha 1, collagen III alpha 1 and Tgfb antibodies were from Novus Biologicals (Bio-Techne GmbH, Wiesbaden-Nordenstadt, Germany).

### Histology

The adipocyte size distribution was determined in formalin-fixed, paraffin-embedded tissues, which were cut into 5 µm sections and stained with hematoxylin and eosin (Carl Roth, Karlsruhe, Germany). Analysis was done with ImageJ using the ADIPOSOFT tool (Schneider et al. 2012). Sirius Red (Direct Red 80; Sigma, Taufkirchen, Germany) staining was done for 30 min using rehydrated tissues which were afterwards dehydrated as explained before (Haberl et al. 2018). Sirius Red staining was quantified by ImageJ (Schneider et al. 2012). Tissue slides were photographed and representative images are shown.

### Monitoring of gene expression

The mRNA expression of murine *Ccn2*, *Bambi*, *Tgfb*, *Acta2*, *Col1a1* and *18S* rRNA was determined by semi-quantitative real-time PCR using SYBR Green (Roche, Mannheim, Germany) as described (Bauer et al. 2011). Total cellular RNA was isolated with TRIzol reagent from GIBCO (Carlsbad, CA) and 1 µg RNA was reverse transcribed using the Promega Reverse Transcription System (Promega, Madison, WI) in a volume of 40 µl; 2 µl of the cDNA was used for amplification in glass capillaries (LightCycler) using PCR primers specific for murine *Ccn2* (5′ CAA AGC AGC TGC AAA TAC CA 3′ and 5′ GGC CAA ATG TGT CTT CCA GT 3′), *Bambi* (5′ CCA AGA GCG AAG CCT CAG 3′ and 5′ AAT GGG AAC CGC TAT CAC AG 3′), *Tgfb* (5′ CTG GGC ACC ATC CAT GAC 3′ and 5′ CAG TTC TTC TCT GTG GAG CTG A 3′), *Acta2* (5′ CCA GCA CCA TGA AGA TCA AG 3′ and 5′ CTT CGT CGT ATT CCT GTT TGC 3′), *Col1a1* (5′ GAC TGG CAA CCT CAA GAA GG 3′ and 5′ CAA GTT CCG GTG TGA CTC G 3′) and *18S* rRNA (5′ GAT TGA TAG CTC TTT CTC GAT TCC 3′ and 5′ CAT CTA AGG GCA TCA CAG ACC 3′). These oligonucleotides were synthesized by Metabion (Planegg-Martinsried, Germany). For quantification of the results, RNA isolated from adipose tissues was reverse transcribed, cDNA was serially diluted and analyzed for the expression of the respective gene to obtain a standard curve. Values were normalized to *18S* rRNA expression.

The Mouse Gene 2.1. ST Array (Affymetrix) was hybridized with total RNA from subcutaneous fat of 5 WT mice kept on SD and 5 WT mice fed a HFD as described above. Sample processing and Affymetrix microarray hybridization were carried out at the Genomics Core Unit: Center of Excellence for Fluorescent Bioanalytics [Kompetenzzentrum für Fluoreszente Bioanalytik (KFB), University of Regensburg, Germany] (Eisinger et al. 2018).

### Statistical analysis

Data are shown as box plots (median, upper and lower quartile, and the largest and lowest value in the data set) or as bar charts (mean value ± standard deviation) (IBM SPSS Statistics 26). Statistical analysis was done by Kruskal–Wallis-test, Mann–Whitney *U* test (IBM SPSS Statistics 26) or Student’s *t* test (Microsoft Excel). A value of *p* < 0.05 was regarded as significant.

## Results

### Expression of *Ccn2*, *Tgfb*, *Acta2*, *Bambi* and *Col1a1* in different fat depots of mice fed a standard diet (SD)

In mice, white adipose tissues are organized as subcutaneous and intra-abdominal fat depots. Brown adipose tissue (BAT) markedly differs from white fat (Sanchez-Gurmaches and Guertin 2014; Kajimura et al. 2015). Whether the expression of genes with a role in fibrosis also varies between the different fat depots has not been studied in very detail so far.

**Fig. 1:**
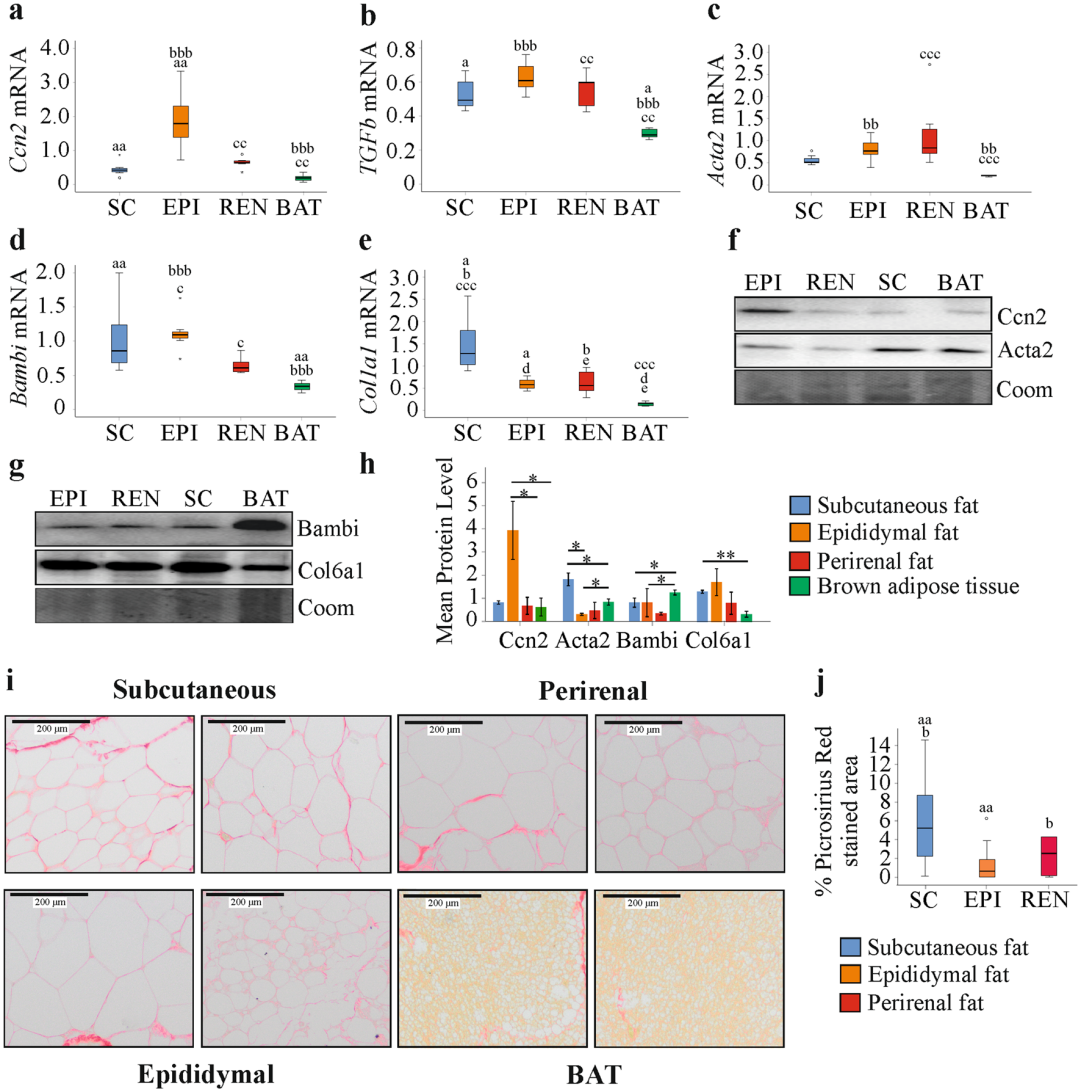
Expression of *Ccn2*/Ccn2, *Tgfb*, *Acta2/*Acta2, *Bambi/*Bambi, *Col1a1* and Col6a1 in subcutaneous (SC), epididymal (EPI), perirenal (REN) and brown adipose tissue (BAT) from 21-week-old mice fed a standard diet. Expression of **a** *Ccn2* **b** *Tgfb* **c** *Acta2* **d** *Bambi* and **e** *Col1a1* in fat tissues. Identical letters in the figures indicate significantly different expression between these two tissues. *p* < 0.05 (one letter), *p* < 0.01 (two letters), *p* < 0.001 (three letters) (*n* = 7 per group). **f** Immunoblot of Ccn2 and Acta2 in these tissues. **g** Immunoblot of Bambi and Col6a1 in these tissues. **h** Quantification of Ccn2, Acta2, Bambi and Col6a1 in the fat tissues of 3 mice. *p < 0.05, **p < 0.01. **i** Sirius Red stained fat tissues. **j** % Picrosirius red stained area of subcutaneous, epididymal and perirenal fat. Because of the brownish color of BAT, this tissue was not suitable for ImageJ analysis

In fat tissue of SD fed mice, *Ccn2* mRNA was highest in epididymal fat in comparison to the other adipose tissues, the difference between epididymal and perirenal fat was not significant (Fig. 1a). The expression of *Tgfb* mRNA did not differ between the white fat tissues and was lowest in BAT in comparison to all white fat tissues (Fig. 1b). *Acta2* mRNA was higher in epididymal and perirenal fat compared to BAT (Fig. 1c). *Bambi* was low expressed in perirenal fat and BAT (Fig. 1d). This was significant for the comparison of epididymal and perirenal fat, epididymal fat and BAT, and subcutaneous fat and BAT (Fig. 1d). *Col1a1* mRNA was highly expressed in subcutaneous fat in comparison to the other fat tissues, and was lowest in BAT (Fig. 1e).

### Expression of Ccn2, Acta2, Bambi and Col6a1 in different fat depots of mice fed a standard diet (SD)

Immunoblot experiments using tissues of three animals showed that Ccn2 protein was high in epididymal adipose tissue and Acta2 protein was high in subcutaneous fat compared to the three other fat depots (Fig. 1f, h). Bambi protein was most abundant in BAT (Fig. 1g, h). Col1a1, Col3a1 and Tgfb were hardly detectable by immunoblot analysis, and data could not be quantified (data not shown). Collagen 6 comprises Col6a1, Col6a2 and Col6a3 chains (Pasarica et al. 2009). *Col6a1* was found induced in epididymal adipose tissue of C57BL/6 mice fed a HFD suggesting a role in adipose tissue fibrosis (Marcelin et al. 2017), and here Col6a1 protein was analyzed. Col6a1 was low expressed in BAT and was comparably abundant in the white fat depots (Fig. 1g, h). Picrosirius red staining was used to visualize collagen fibers. Collagen deposition around adipocytes was more prominent in subcutaneous fat (Fig. 1i). Fibrous bundles were detected in the subcutaneous, perirenal and BAT depots of all mice and in epididymal fat of about half of the mice (Fig. 1i and data not shown). Quantification of picrosirius red stained areas in the white fat depots revealed that % stained area was higher in subcutaneous compared to perirenal and epididymal fat (Fig. 1j). BAT staining was not quantified because of the brownish color of these tissues.

Adipocyte area was found associated with increased collagen expression (Sun et al. 2011, 2013). Adipocyte area was, however, highest in perirenal fat and did not differ between subcutaneous and epididymal adipose tissues (Supporting Fig. 1).

### Comparison of *Ccn2/*Ccn2, *Bambi/*Bambi, *Tgfb, Acta2*/Acta2 and *Col1a1* expression in different fat depots of mice fed a SD or a high fat diet (HFD)

Feeding mice a HFD causes adipocyte dysfunction and can induce adipose tissue fibrosis (Buechler et al. 2015; Sun et al. 2013; Kwon and Pessin 2012). The mice fed a HFD for 14 weeks had increased body weight, higher serum triglyceride and aspartate aminotransferase levels (Table 1). Fasting glucose was not significantly induced (Table 1). Adipocyte volume was determined in epididymal fat and subcutaneous fat and was increased in the obese animals (Supporting Fig. 2a, b).

**Table 1:** Final body weight, blood glucose, triglycerides and aspartate aminotransferase of the 21-week-old mice fed a standard diet (SD) or high fat diet (HFD) for the last 14 weeks (median value and range are given)

Measure	SD	HFD	*p*-value
Body weight (g)	26.1 (25.4–30.7)	36.2 (31.4–44.8)	0.002
Glucose (mg/dl)	168 (116–270)	242 (189–336)	0.073
Triglycerides (mg/dl)	105 (88–144)	130 (105–189)	0.029
Aspartate aminotransferase (U/l)	77 (56–154)	144 (77–210)	0.030

**Fig. 2:**
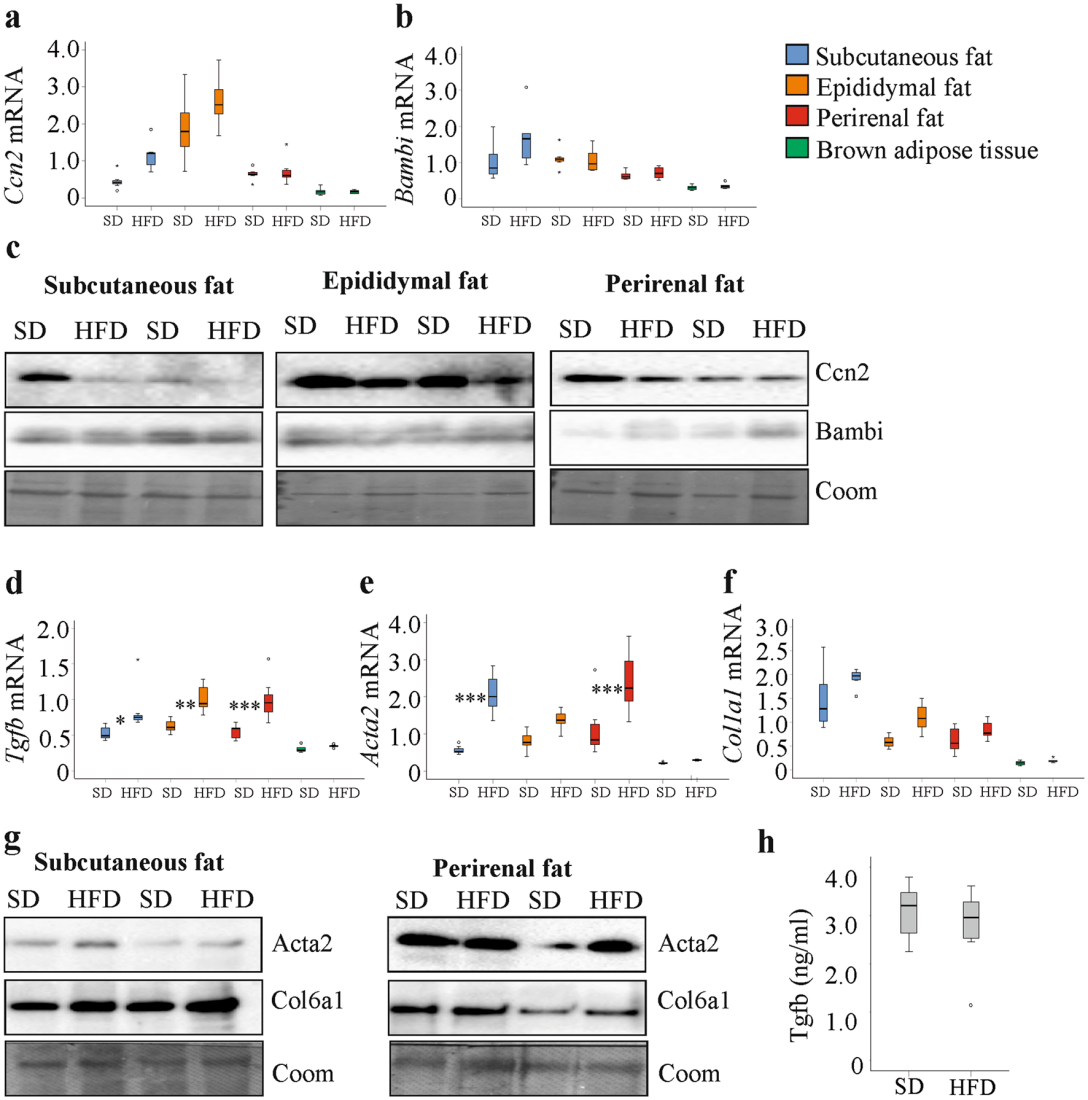
Expression of fibrosis-related genes and proteins in fat tissue of mice fed a standard diet (SD) or a high fat diet (HFD). **a** *Ccn2* mRNA in the different fat depots of mice fed a SD or HFD. **b** *Bambi* mRNA in these tissues. **c** Ccn2 and BAMBI protein in the different white fat depots of two mice fed a SD and two mice fed a HFD. Coomassie (Coom) stained membrane is shown as loading control. **d** *Tgfb*, **e** *Acta2* and **f** *Col1a1* mRNA in the different fat depots of mice fed a SD or HFD. **g** Acta2 and Col6a1 protein in subcutaneous and perirenal fat of two mice fed a SD and two mice fed a HFD. **h** Tgfb protein in the serum of these mice *p < 0.05, **p < 0.01, ***p < 0.001 for comparison of gene expression between SD and HFD fed mice (*n* = 7 per group for mRNA expression analysis, *n* = 6 per group for immunoblot analysis and *n* = 5 per group for data shown in (**h**)

*Ccn2* and *Bambi* mRNA were not upregulated in the fat depots of the mice fed a HFD (Fig. 2a, b). Immunoblot analysis showed that Ccn2 and Bambi protein were similar in the white fat depots of SD and HFD fed animals (Fig. 2c and Table 2).

**Table 2:** Ccn2 and Bambi protein in subcutaneous (sc), epididymal (epi) and perirenal fat and Acta2 und Col6a1 protein in subcutaneous and perirenal fat of 21-week-old mice, which were fed a standard diet (SD) or high fat diet (HFD) for the last 14 weeks (median values and range are given)

	SD sc	HFD sc	SD epi	HFD epi	SD ren	HFD ren
Ccn2	0.20 (0.10–0.72)	0.12 (0.03–0.49)	1.92 (1.18–3.48)	1.31 (0.68–2.42)	4.38 (2.52–6.02)	3.53 (1.37–5.81)
Bambi	0.45 (0.36–0.71)	0.56 (0.39–1.06)	1.01 (0.13–1.94)	1.29 (0.11–3.93)	3.91 (0.52–4.81)	4.54 (2.76–8.52)
Acta2	0.39* (0.26–0.60)	3.18* (1.01–5.04)	Not determined	Not determined	0.34* (0.15–1.66)	2.74* (0.73–4.22)
Col6a1	1.98* (0.95–2.20)	2.44* (2.06–5.26)	Not determined	Not determined	1.22 (0.91–1.40)	2.20 (1.09–3.16)

*p < 0.05

*Tgfb* mRNA was induced in all of the white fat depots, and *Acta2* mRNA in subcutaneous and perirenal fat of the overweight mice (Fig. 2d, e). *Col1a1* mRNA levels did not significantly change in the adipose tissues of the animals fed the HFD (Fig. 2f).

*Acta2* mRNA was induced in subcutaneous and perirenal fat of HFD fed mice (Fig. 2e), and immunoblot experiments showed that Acta2 protein was higher in these adipose tissues (Fig. 2g and Table 2). Col6a1 was also analyzed, and was increased in subcutaneous adipose tissues of HFD fed mice (Fig. 2g and Table 2). Tgfb was measured in serum by ELISA and was similar in both groups (Fig. 2h).

Analysis of *Ccn2*, *Bambi*, *Col1a1*, *Col6a1*, *Tgfb* and *Acta2* mRNAs in subcutaneous fat of 28-week-old mice, which were either fed a SD or a HFD for the last 14 weeks, by GeneChip analysis showed an upregulation of *Acta2* mRNA whereas the expression of all other genes did not change (Supporting Fig. 2c).

To find out whether the tissue-specific expression of these genes was changed in the overweight animals, mRNA levels in the different fat depots of the mice fed a HFD were compared. Here, *Ccn2* was highest expressed in epididymal fat as was found in the SD fed animals (Fig. 1a and Supporting Fig. 3a). *Tgfb* mRNA was higher in the white fat depots compared to BAT with no differences between the white fat depots, and this was observed in the HFD and SD fed animals (Fig. 1b and Supporting Fig. 3b). *Acta2* mRNA was low abundant in BAT of SD and HFD fed animals. A difference between perirenal and epididymal levels was only found in the overweight mice (Fig. 2c and Supporting Fig. 3c). *Bambi* was higher expressed in subcutaneous and epididymal fat compared to BAT, and this distribution was observed in SD and HFD fed animals (Fig. 1d and Supporting Fig. 3d). *Col1a1* mRNA was highest in subcutaneous fat. The mRNA levels in BAT were low in comparison to perirenal and epididymal fat of SD and HFD fed mice (Fig. 1e and Supporting Fig. 3e).

### Correlation of *Ccn2*, *Bambi*, *Tgfb*, *Acta2* and *Col1a1* in different fat depots with body weight, fasting glucose and *adhesion G protein-coupled receptor E1* (*Adgre*, coding for the cell surface glycoprotein F4/80) mRNA

Associations of the expression of fibrotic genes in fat tissues with body weight and fasting glucose were described in humans (Yoshino et al. 2019). In the group of mice fed either a SD or a HFD, subcutaneous, epididymal and perirenal *Tgfb* mRNA levels as well as subcutaneous and epididymal *Acta2* mRNA levels were positively correlated with body weight. Such an association also existed for epididymal *Col1a1* mRNA (Table 3). Perirenal *Tgfb*, epididymal and perirenal *Col1a1* positively correlated with blood glucose (Table 3 and Supporting Fig. 4a, b).

**Table 3:** Spearman correlation coefficients and *p*-values for significant correlations of gene expression with body weight and blood glucose in mice fed a SD or HFD

Measure	Sc *Tgfb*	Epi *Tgfb*	Ren *Tgfb*	Sc *Acta2*	Epi *Acta2*	Epi *Col1a1*	Ren *Col1A1*
*SD and HFD fed mice*							
Body weight							
*r*	0.736	0.793	0.837	0.780	0.763	0.886	
*p*	0.013	0.005	0.001	0.005	0.010	< 0.001	
Glucose							
*r*	–	–	0.722	–	–	0.685	0.870
*p*			0.018			0.034	< 0.001
*HFD fed mice*							
Body weight							
*r*		0.964	–	–	–	–	
*p*		< 0.001					
Glucose							
*r*			0.786			–	–
*p*			0.036				

*Epi* epididymal, *Ren* perirenal, *Sc* subcutaneous

Macrophages, which express *Adgre*, accumulate in fat tissues in obesity (Hill et al. 2014). *Adgre* mRNA was positively correlated with subcutaneous and perirenal *Tgfb* mRNA (Supporting Fig. 4c, d). In the group of HFD mice no significant associations existed (data not shown) suggesting that these correlations are related to the differences between the SD and HFD fed mice.

In the HFD group epididymal *Tgfb* mRNA still positively correlated with body weight, and perirenal *Tgfb* mRNA with blood glucose (Table 3).

### Expression of *Ccn2/*Ccn2, *Bambi/*Bambi, *Tgfb*, *Acta2*/Acta2, *Col1a1* and Col6a1 in different fat depots of control animals and ob/ob mice

Leptin-deficient mice are extremely obese (Table 4) and adipocyte area, which was determined in perirenal and subcutaneous fat, was much larger in the ob/ob animals (Supporting Fig. 5a, b). Serum triglycerides and cholesterol of the ob/ob mice were markedly higher in comparison to the lean WT animals (Table 4).

**Table 4:** Body weight, blood triglycerides and cholesterol of wild type (WT) and ob/ob mice (median value and range)

Measure	WT	ob/ob	*p*-value
Body weight (g)	23.2 (21.6–27.4)	51.6 (47.4–55.4)	0.009
Triglycerides (mg/dl)	128 (122–131)	156 (150–259)	0.009
Cholesterol (mg/dl)	53 (51–61)	107 (78–127)	0.009

**Fig. 3:**
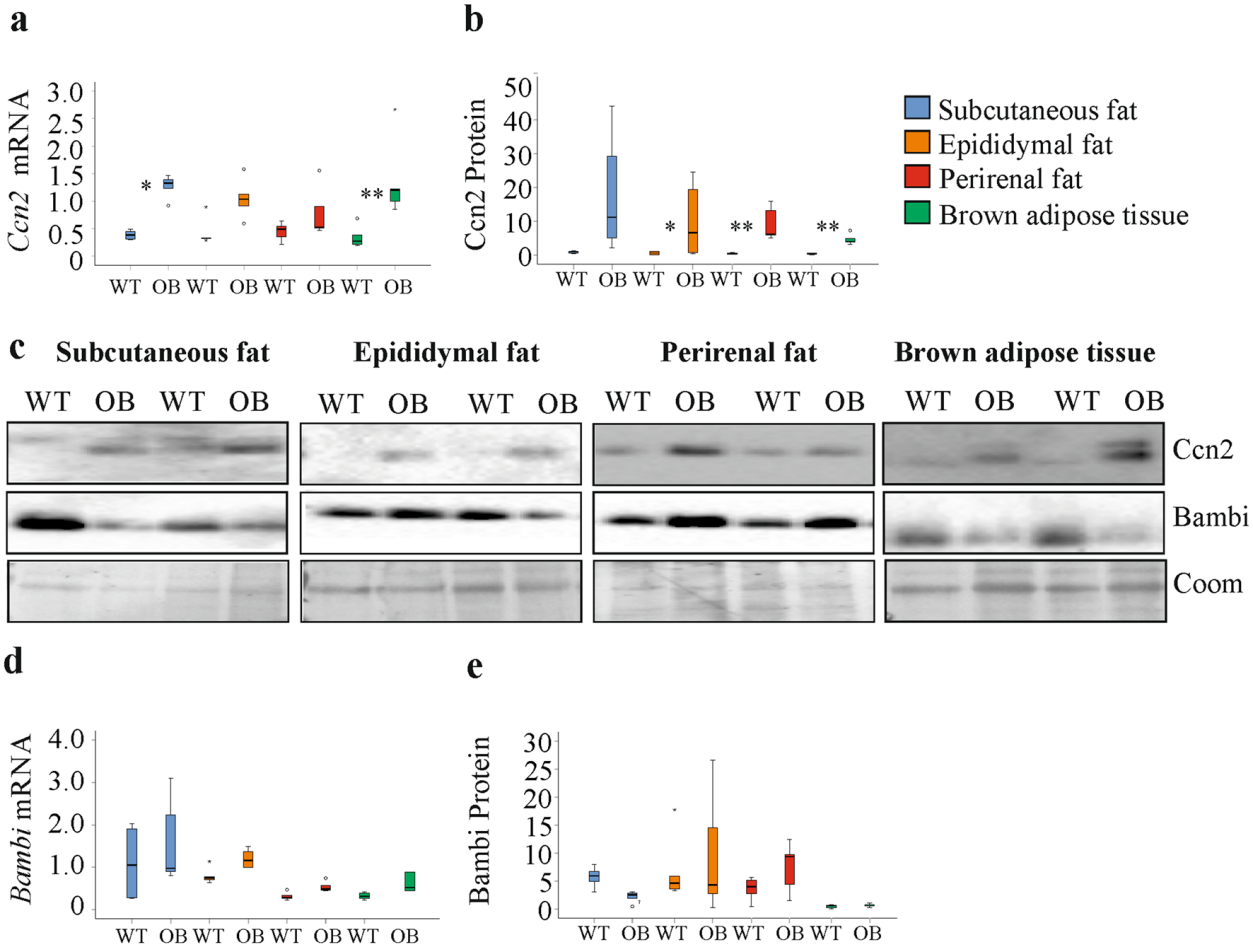
Expression of *Ccn2*/Ccn2 and *Bambi*/Bambi in fat tissue of wild type (WT) and ob/ob (OB) mice. **a** *Ccn2* mRNA in the different fat depots. **b** Quantification of Ccn2 protein in the different fat depots. **c** Immunoblot of Ccn2 and Bambi protein in the different fat depots of two WT and 2 OB mice. Coomassie (Coom) stained membrane is shown as loading control. **d** *Bambi* mRNA in the different fat depots. **e** Quantification of Bambi protein in the different fat depots of mice. *p < 0.05, **p < 0.01 for comparison of gene expression between ob/ob and WT mice (*n* = 5 per group)

*Ccn2* mRNA was increased in subcutaneous fat and BAT of the ob/ob mice in comparison to the respective control animals (Fig. 3a). Ccn2protein was induced in the white fat tissues and BAT, and this was significant for all but subcutaneous fat (Fig. 3b, c). *Bambi* mRNA and Bambi protein were not changed in the obese adipose tissues (Fig. 3c, d, e).

**Fig. 4:**
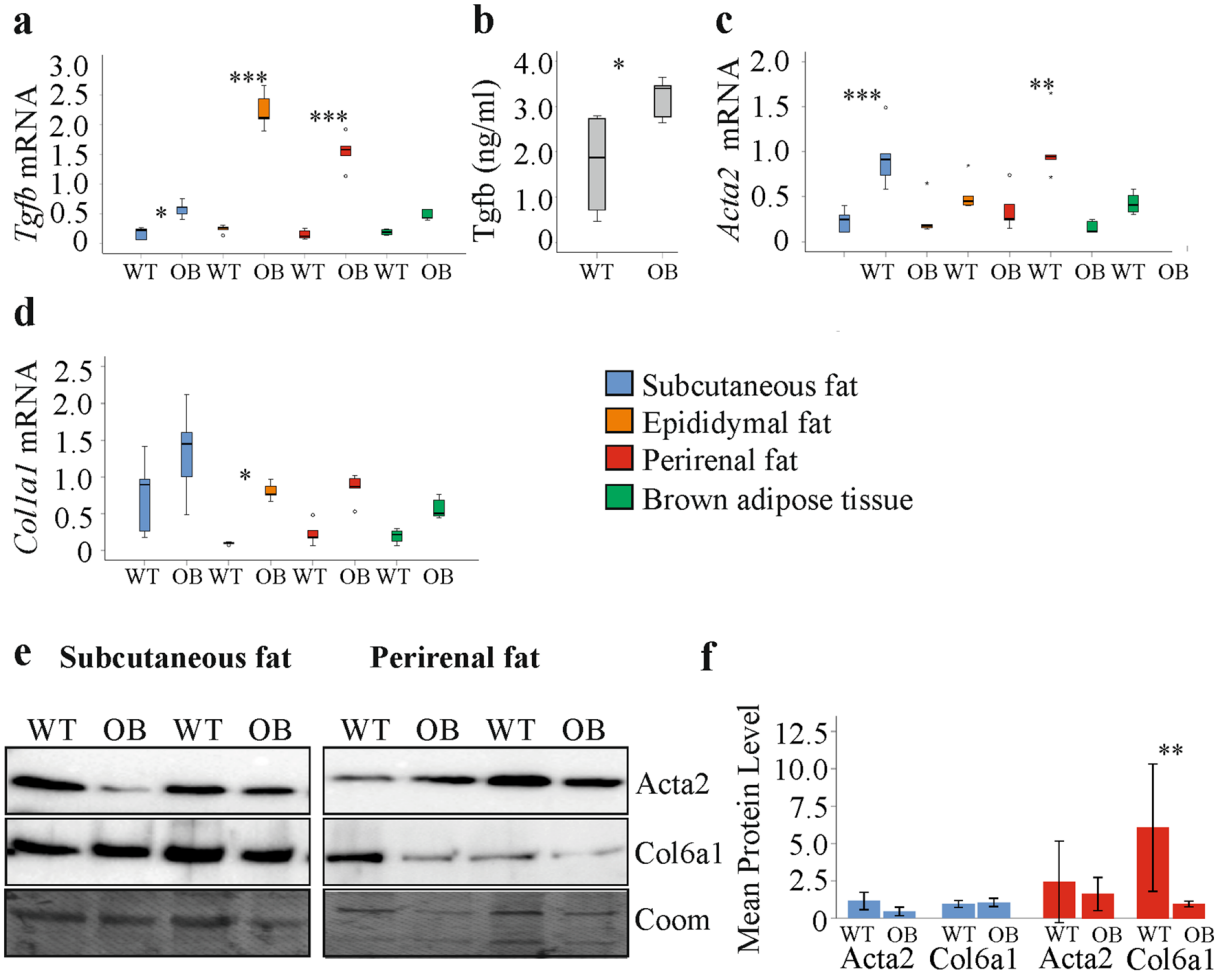
Serum Tgfb and expression of *Tgfb*, *Acta2*/Acta2, *Col1a1* and Col6a1 in fat tissue of wild type (WT) and ob/ob (OB) mice. **a** *Tgfb* mRNA in the different fat depots. **b** Serum Tgfb of WT and ob/ob mice. **c** *Acta2* mRNA in the different fat depots. **d** *Col1a1* mRNA in the different fat depots. **e** Acta2 and Col6a1 protein in subcutaneous and perirenal fat of two WT and two ob/ob mice. **f** Quantification of Acta2 and Col6a1 protein in subcutaneous and perirenal fat. (*n* = 5 per group). *p < 0.05, **p < 0.01, ***p < 0.001 for comparison of gene/protein expression between ob/ob and WT mice

*Tgfb* mRNA was induced in subcutaneous, epididymal and perirenal fat of the ob/ob animals (Fig. 4a). Notably, Tgfb in serum was also higher (Fig. 4b). *Acta2* mRNA was induced in subcutaneous and perirenal fat of the ob/ob animals, and *Col1a1* upregulation was significant in epididymal fat (Fig. 4c, d). Immunoblot analysis did not detect higher Acta2 protein in subcutaneous and perirenal fat of ob/ob mice (Fig. 4e, f). Col6a1 protein was not changed in the subcutaneous fat depots of the obese animals and was reduced in perirenal fat of ob/ob mice (Fig. 4e, f).

Comparison of the different fat tissues of the ob/ob mice showed that *Ccn2* expression was similar in all of them (Supporting Fig. 6a). *Tgfb* was higher in epididymal and perirenal fat than brown adipose tissue, and in epididymal fat compared to subcutaneous fat (Supporting Fig. 6b). *Acta2* was comparable in epididymal and brown fat. Expression was higher in subcutaneous and perirenal fat in comparison to epididymal fat and BAT (Supporting Fig. 6c). *Bambi* mRNA was principally lower expressed in perirenal fat and BAT (Supporting Fig. 6d). *Col1a1* only differed between subcutaneous and brown fat, and was lower in the latter (Supporting Fig. 6e).

### Effect of fatty acids, leptin and lipopolysaccharide on *Ccn2*, *Bambi* and *Tgfb* mRNA and Ccn2 protein expression in 3T3-L1 cells

**Fig. 5:**
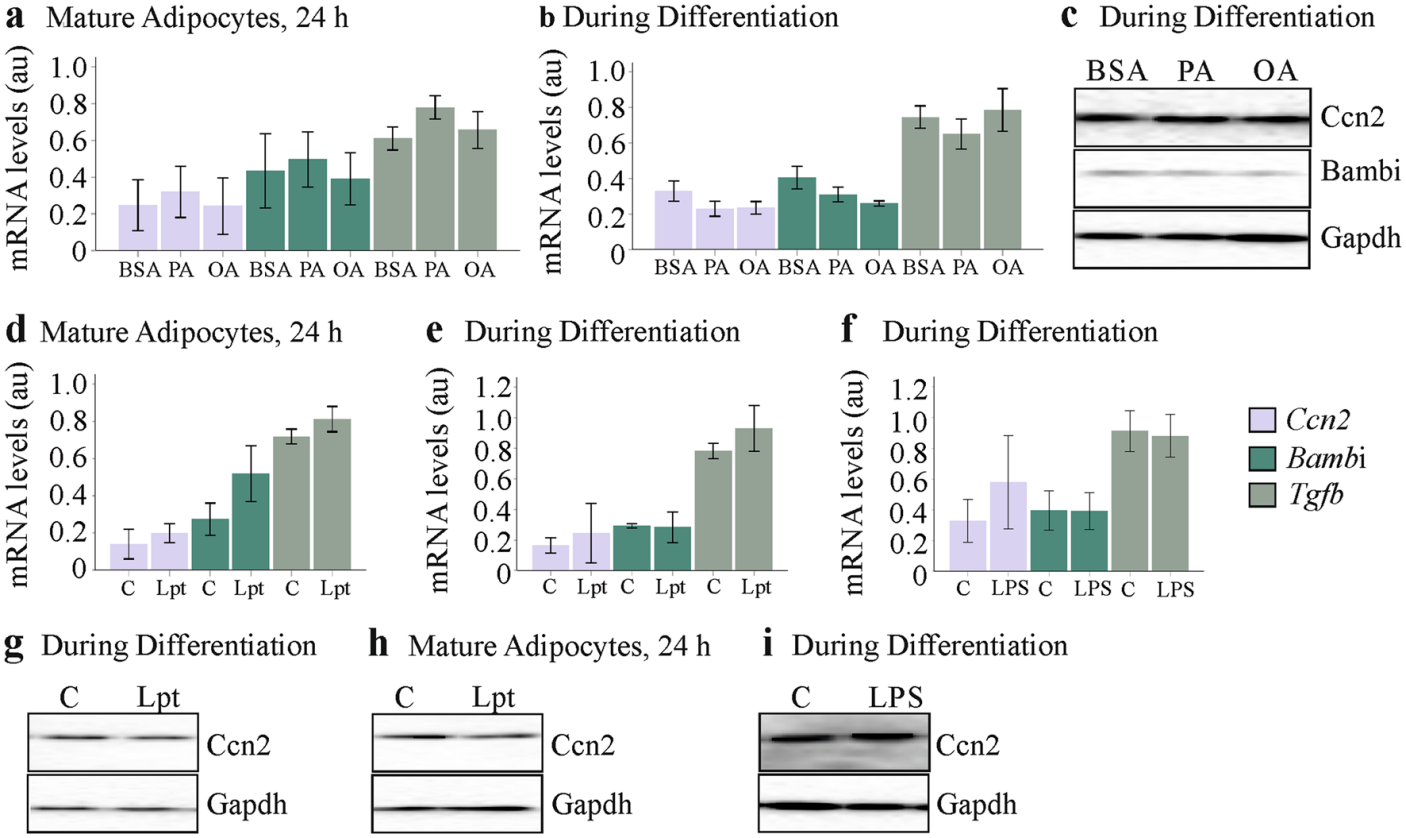
Effect of palmitic acid (PA), oleic acid (OA), leptin (Lpt) and lipopolysaccharide (LPS) on *Ccn2*, *Bambi* and *Tgfb* expression and on Ccn2 protein levels of 3T3-L1 cells. **a** Expression of these genes in 3T3-L1 adipocytes which were incubated with 100 µM fatty acids for 24 h after differentiation to mature cells (*n* = 3). **b** Expression of these genes in 3T3-L1 adipocytes, which were differentiated in the presence of 100 µM fatty acids (*n* = 3). **c** Immunoblot of Ccn2 and Bambi in the cells described in (**b**). **d** Expression of these genes in 3T3-L1 adipocytes, which were incubated with 100 ng/ml leptin for 24 h after differentiation to mature cells. **e** Expression of these genes in 3T3-L1 adipocytes, which were differentiated in the presence of 100 ng/ml leptin (*n* = 3). **f** Expression of these genes in 3T3-L1 adipocytes, which were differentiated in the presence of 10 ng/ml LPS (*n* = 6). **g** Immunoblot of Ccn2 in 3T3-L1 cells differentiated in the presence of 100 ng/ml leptin. **h** Immunoblot of Ccn2 in 3T3-L1 adipocytes, which were incubated with 100 ng/ml leptin for 24 h after differentiation to mature cells. **i** Immunoblot of Ccn2 in 3T3-L1 cells differentiated in the presence of 10 ng/ml LPS. Arbitrary units, au

Adipose tissues are composed of different cells and higher expression of distinct genes in the obese may be related to altered cellular composition and/or upregulation of these genes in different cells such as adipocytes (Buechler et al. 2015). The 3T3-L1 cell line is a commonly used model of white adipocytes (Morrison and McGee 2015). Cultivation of these cells in media supplemented with fatty acids promotes triglyceride deposition (Krautbauer et al. 2014a, b). Palmitic acid and oleic acid supplementation of mature cells or when added during differentiation did, however, not affect *Ccn2*, *Bambi* and *Tgfb* mRNA levels (Fig. 5a, b). Ccn2 and Bambi protein were not upregulated in these cells (Fig. 5c). Stimulation of mature 3T3-L1 adipocytes with leptin (100 ng/ml) for 24 h had no effect on the expression of these genes (Fig. 5d). LPS (10 ng/ml) or leptin (100 ng/ml) added to cell media during differentiation of 3T3-L1 cells did not change *Ccn2*, *Bambi* or *Tgfb* mRNA levels (Fig. 5e, f). Immunoblot experiments could not identify an effect of leptin (added for 24 h or during differentiation) or LPS (added during differentiation) on Ccn2 protein levels (Fig. 5g–i).

### Effect of fatty acids, leptin, LPS and inflammatory cytokines on Tgfb levels in cell media

**Fig. 6:**
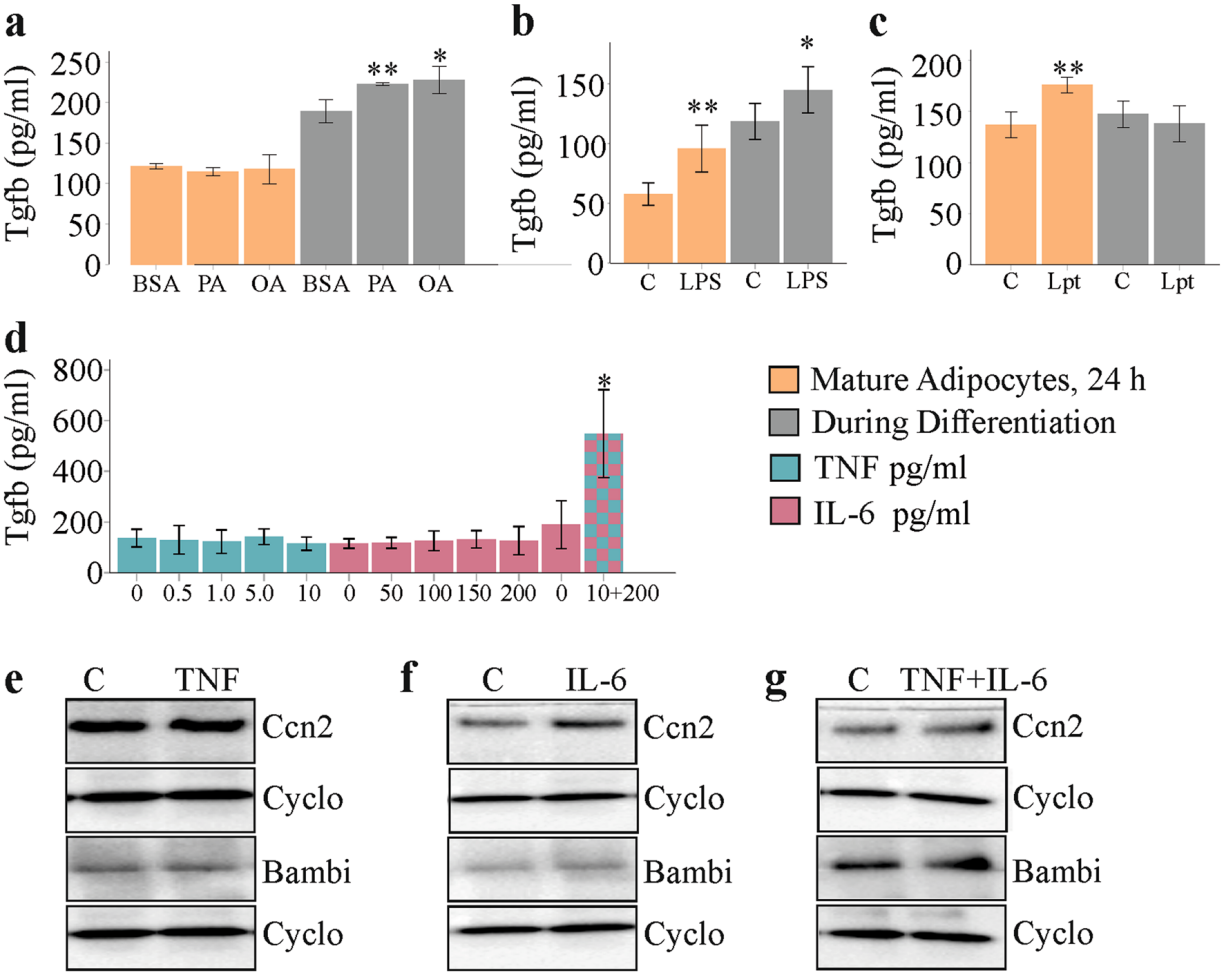
Effect of palmitic acid (PA), oleic acid (OA), leptin (Lpt), lipopolysaccharide (LPS), TNF and IL-6 on Tgfb, Ccn2 and Bambi protein levels of 3T3-L1 cells. **a** Tgfb in cell media of 3T3-L1 adipocytes, which were incubated with fatty acids for 24 h after differentiation to mature cells (*n* = 4) or were differentiated in the presence of 100 µM fatty acids (*n* = 6). **b** Tgfb in cell media of 3T3-L1 adipocytes, which were incubated with LPS for 24 h after differentiation to mature cells (*n* = 6) or were differentiated in the presence of 10 ng/ml LPS (*n* = 6). **c** Tgfb in cell media of 3T3-L1 adipocytes, which were incubated with leptin for 24 h after differentiation to mature cells (*n* = 4) or were differentiated in the presence of 100 ng/ml leptin (*n* = 3). **d** Tgfb in cell media of 3T3-L1 adipocytes, which were differentiated in the presence of TNF, IL-6 or both (*n* = 3). **e** Immunoblot of Ccn2 and Bambi in 3T3-L1 adipocytes, which were differentiated in the presence of 10 pg/ml TNF. **f** Immunoblot of Ccn2 and Bambi in 3T3-L1 adipocytes, which were differentiated in the presence of 200 pg/ml IL-6. **g** Immunoblot of Ccn2 and Bambi in 3T3-L1 adipocytes, which were differentiated in the presence of 10 pg/ml TNF and 200 pg/ml IL-6. Cyclophilin A (Cyclo) was used as loading control

Tgfb is a central molecule in fibrogenesis (Lee 2018) and was measured in the supernatants of 3T3-L1 cells. Tgfb protein was induced in media of cells differentiated in the presence of 100 µM PA or OA. Fatty acids did not increase Tgfb in 3T3-L1 adipocytes during 24 h cultivation (Fig. 6a). Tgfb in cell media was higher in differentiated cells upon 24 h stimulation with LPS and when LPS was added to the cells during differentiation (Fig. 6b). Leptin induced Tgfb only in the differentiated adipocytes and had no effect during adipogenesis (Fig. 6c). IL-6 (50, 100, 150 and 200 pg/ml) and TNF (0.5, 1.0, 5.0 and 10 pg/ml), when added during 3T3-L1 cell adipogenesis were inactive with regard to Tgfb upregulation. When both, TNF (10 pg/ml) and IL-6 (200 pg/ml), were added to the cell media during 3T3-L1 differentiation, a marked increase of Tgfb was observed (Fig. 6d).

Ccn2 could not be detected in the 3T3-L1 cell media by an ELISA with a detection limit of 31.2 pg/ml. Ccn2 and Bambi protein were not regulated in these cells upon differentiation in the presence of 10 pg/ml TNF, 200 pg/ml IL-6 or both (Fig. 6e–g). Col1a1 protein could not be detected in the 3T3-L1 cells by immunoblot analysis (data not shown).

**Fig. 7:**
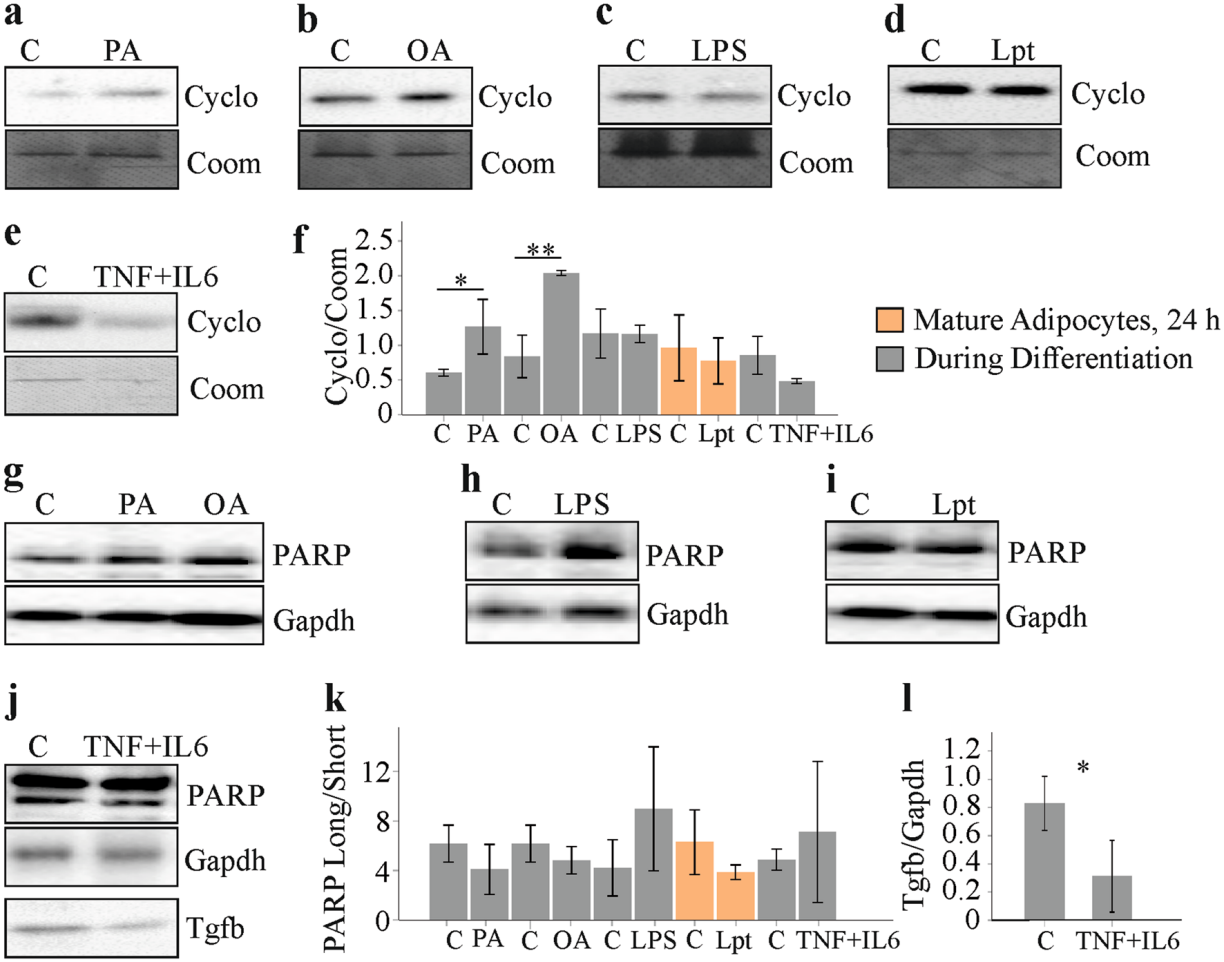
Cell death markers in 3T3-L1 cells treated with palmitic acid (PA), oleic acid (OA), leptin (Lpt), lipopolysaccharide (LPS), TNF and IL-6. **a** Cyclophilin A (Cyclo) in media of cells differentiated in the presence of PA. **b** Cyclophilin A (Cyclo) in media of cells differentiated in the presence of OA. **c** Cyclophilin A (Cyclo) in media of mature cells incubated with LPS for 24 h. **d** Cyclophilin A (Cyclo) in media of mature cells incubated with leptin for 24 h. **e** Cyclophilin A in cell media of 3T3-L1 adipocytes, which were differentiated in the presence of TNF and IL-6. **f** Quantification of cyclophilin A in cell media (*n* = 3). *p < 0.05, **p < 0.01. **g** PARP in cells differentiated in the presence of PA or OA. **h** PARP in cells differentiated in the presence of LPS. **i** PARP in cells incubated with leptin for 24 h. **j** PARP in 3T3-L1 adipocytes, which were differentiated in the presence of TNF and IL-6. **k** Ratio of uncleaved to cleaved PARP (*n* = 3). **l** Tgfb in 3T3-L1 adipocytes, which were differentiated in the presence of TNF and IL-6

Necrotic adipocytes release their intracellular contents (Wagner 2014) and this may contribute to higher Tgfb in cell media. Cyclophilin A is released early in necrosis (Christofferson and Yuan 2010), and was quantified in cell supernatants. Cells differentiated in the presence of PA or OA indeed had higher cyclophilin A in cell media (Fig. 7a, b, f). A rise could not be observed in cells differentiated in the presence of LPS or incubated with leptin for 24 h (Fig. 7c, d, f). Differentiation of 3T3-L1 cells in the presence of TNF and IL-6 did not change cyclophilin A in cell media (Fig. 7e, f).

Apoptotic cells can release Tgfb, and here, *Tgfb* transcription is not induced (Chen et al. 2001). Poly(ADP-ribose) polymerase-1 (PARP-1) is cleaved by caspases in apoptotic cells (Putt et al. 2005). Cleavage of this protein was, however, not changed in 3T3-L1 cells differentiated in the presence of PA or OA (Fig. 7g, k) or LPS (Fig. 7h, k) and was not enhanced when mature 3T3-L1 adipocytes were incubated with leptin for 24 h (Fig. 7i, k). Differentiation of 3T3-L1 cells in the presence of TNF and IL-6 did not induce PARP cleavage, but lowered endogenous Tgfb protein level (Fig. 7j, l).

## Discussion

This study showed that fat depot related characteristics in the expression of fibrosis-related genes are mostly conserved in obesity. Upregulation of *Tgfb* mRNA in obese white fat is not consistently accompanied by higher expression of its downstream genes suggesting that Tgfb activity differs between the adipose tissue depots. Obesity-related increase of *Acta2* mRNA in both animal models was associated with higher Acta2 protein levels in the HFD model but not the ob/ob mice illustrating that posttranslational regulation of Acta2 expression varies between these two models (Supporting Fig. 7).

White and brown fat differ in various aspects, among others BAT contains more mitochondria and numerous small lipid droplets (Sanchez-Gurmaches and Guertin 2014; Kajimura et al. 2015). The pro-fibrotic genes *Tgfb*, *Ccn2, Acta2* and *Col1a1* were low expressed in BAT in comparison to the white adipose tissues. Analysis of Col6a1 protein also revealed low abundance in BAT in comparison to the white fat depots. Col6a1 protein was comparable in the different white fat tissues, and similar mRNA expression in inguinal, epididymal, mesenteric and perirenal fat of mice has been shown before (Khan et al. 2009).

Subcutaneous and visceral fat differ in cellularity, innervation and cellular composition (Ibrahim 2010). There is convincing evidence that excess visceral adiposity is a risk factor for insulin resistance, chronic inflammation, nonalcoholic steatohepatitis and cardiovascular diseases (Ronn et al. 2020; Schaffler et al. 2005; Janochova et al. 2019). Rodent epididymal and perirenal adipose tissues are commonly used intra-abdominal fat depots to study the harmful effects of excess adiposity in obesity (Ibrahim 2010; Altintas et al. 2011; Krautbauer et al. 2019).

*Ccn2* mRNA was highest in epididymal fat. Mice fed a regular chow had about twofold increased *Ccn2* mRNA level in epididymal compared to subcutaneous fat (Tan et al. 2008) in accordance with the current analysis. Immunoblot showed that Ccn2 protein is higher in epididymal fat in comparison to subcutaneous, perirenal and brown adipose tissues. Ccn2 protein levels did, however, not differ between subcutaneous fat or perirenal fat and BAT though mRNA expression was lowest in BAT.

Acta2 protein was highest in subcutaneous fat, and low expression in BAT, as was indicated by the mRNA data, could not be detected at the protein level.

*Bambi* mRNA was similar in perirenal fat and BAT, and expression in these fat depots was lower than in subcutaneous and epididymal fat. *Bambi* mRNA levels were not consistent with the protein expression, and Bambi protein was high in BAT. Discordant findings regarding protein and mRNA expression of Bambi have been described before (Weber et al. 2023). Hence, it is important to analyze protein expression to obtain reliable information about the expression of Bambi and proteins such as Acta2 in the different fat depots. Typically ~ 30 to 60% of the variance in protein levels can be attributed to mRNA expression. Other variations are explained by post-transcriptional and post-translation mechanisms (Sousa et al. 2009; Vogel and Marcotte 2012). To deduce an altered protein level from mRNA expression data, which is frequently done, may be misleading (Marcelin et al. 2019; Huber et al. 2007; Khan et al. 2009).

Differences in the abundance of the analyzed proteins in the fat tissues may be related to their varying expression in adipose tissue cells and/or differences in the cellular composition of the specific fat depots (Kadiri et al. 2017; Macotela et al. 2012).

*Ccn2* and *Tgfb* mRNA were induced in BAT of ob/ob mice and were similar between subcutaneous fat and BAT of these animals. In these extremely obese animals BAT seems to acquire characteristics of white fat with regard to the expression of these two pro-fibrotic genes. Immunoblot analysis is, however, required to confirm this suggestion.

*Bambi* mRNA levels did not greatly change in obesity. Current findings are in contrast to a study showing that *Bambi* mRNA was low in epididymal fat of ob/ob and HFD fed mice (Luo et al. 2012). A change of Bambi protein was not detected in any fat depot of high fat diet and ob/ob mice indicating that Bambi abundance is not altered in obesity.

*Tgfb* mRNA was induced in all white fat tissues of the overweight and obese mice. Notably, an increase of Tgfb protein in epididymal fat of mice fed a HFD for about 8 weeks was described (Kumar et al. 2019). Notably, serum Tgfb was higher in the ob/ob mice but not the animals fed a high fat diet. This is in accordance with increased serum TGFb described in obese humans (Lee 2018). Whether adipose tissues contribute to blood Tgfb/TGFb protein has to be resolved in the future.

Besides its effect on *Ccn2* expression, TGFb upregulates Acta2 and Col1a1 levels in myofibroblasts (Frangogiannis 2020). Acta2 mRNA levels were quite similar between the white fat depots of control diet fed mice. In the overweight/obese mice, *Acta2* mRNA was induced in all fat depots, and this upregulation was significant for subcutaneous and perirenal fat. Acta2 protein was, accordingly, increased in subcutaneous and perirenal fat of mice fed a HFD. Interestingly, upregulation of Acta2 was not observed in these fat depots of the ob/ob mice.

Expression of Col6a1 also differed between these two models. Col6a1 protein was higher in subcutaneous fat of HFD fed mice but did not change in the ob/ob animals. Here, perirenal Col6a1 declined, which was not observed in the diet-induced obese mice.

Leptin can elevate *Acta2* mRNA and Acta2 protein in fibroblasts (Watanabe et al. 2019), and this may contribute to Acta2 upregulation in the diet-induced obese mice where serum leptin is high (Perez-Echarri et al. 2005). OB/ob mice do not express leptin (Sanches et al. 2015), and this may explain why Acta2 protein is not increased. Moreover, downregulation of *Acta2* mRNA in epididymal fat of ob/ob mice in comparison to controls, which was not accompanied by a change of Acta2 protein, has been also reported (Takahashi et al. 2022). Thus, leptin deficiency alone can not explain the discordant findings between diet-induced obese mice and ob/ob mice.

*Col1a1* was highly expressed in subcutaneous adipose tissue of all the mouse groups studied. Picrosirius red staining indicated that collagen was most abundant in subcutaneous fat. Higher expression of collagen 1 protein in subcutaneous than visceral fat of Wistar rats was described before (Mori et al. 2014). Mice had higher *Col1a1* mRNA in subcutaneous fat compared to epididymal fat and BAT (Gonzalez Porras et al. 2021). In subcutaneous fat of obese women total fibrosis was 0.94 ± 0.39% of the area and in visceral fat 0.74 ± 0.35% of the area (Osorio-Conles et al. 2022) indicating that collagen is also higher in human subcutaneous fat.

*Col1a1* mRNA levels did not significantly increase in the fat depots of the HFD fed mice. A further study observed increased mRNA expression of *Col1a1* in epididymal mouse fat after HFD. Col1a1 protein was, however, reduced in the epididymal fat of HFD fed mice and subcutaneous fat of obese humans (Adapala et al. 2012). *Col1a1* mRNA was found upregulated in the fat tissues of the ob/ob mice and this was significant for epididymal fat. Higher expression of *Col1a1* mRNA was also identified in epididymal fat of db/db mice, where leptin signaling is disturbed (Khan et al. 2009). In the extremely obese mice, all of the white fat tissues had similar levels of *Col1a1* mRNA. Whether this applies to Col1a1 protein and indeed is an indicator of fibrotic remodeling in the fat tissues needs further study (Adapala et al. 2012).

Perirenal *Tgfb* mRNA as well as perirenal *Col1a1* and epididymal *Col1a1* mRNA correlated with blood glucose in mice fed either a SD or HFD. Here, subcutaneous and perirenal *Tgfb* mRNA were positively related to *Adgre* expression. In the HFD group, the association of perirenal *Tgfb* and serum glucose was still significant. Yet correlations do not prove functional associations, and further studies have to confirm a role of perirenal expressed Tgfb with glucose metabolism.

To get further insights into the expression of genes and proteins with a role in fibrosis, the 3T3-L1 cell model was employed. 3T3-L1 adipocytes exposed to LPS, leptin, IL-6 and TNF as well as saturated and monounsaturated fatty acids had higher Tgfb in cell media. This was not accompanied by increased Ccn2 protein showing that Ccn2 is not induced by autocrine Tgfb signaling in these cells.

It has to be noted that *Tgfb* mRNA did not increase in the 3T3-L1 cells upon treatment with fatty acids, LPS or inflammatory cytokines. Upregulation of *Tgfb* mRNA by 1 µg/ml LPS was shown in 3T3-L1 adipocytes (Guo et al. 2020), which is a 100-fold higher LPS concentration than used in the current experiments. Hence, identification of the cells in fat tissues that produce increased *Tgfb* mRNA in obesity and the regulatory pathways involved is a matter of future studies. In adipose tissues, non-fat cells were the main producers of TGFb and only 10% is released by adipocytes suggesting that mRNA expression of stromal vascular cells is changed in the obese (Fain et al. 2005).

Here it is important to note that LPS upregulated Tgfb in cell media of mature adipocytes and when added during differentiation. Leptin was only effective in the mature cells and free fatty acids in the differentiation model. These results show that metabolic active factors differentially affect mature adipocytes and when present during cell maturation.

Tgfb is released from dying cells (Chen et al. 2001). The incubations summarized above did not cause apoptotic cell death and this was shown before for fatty acid treatment by our group (Krautbauer et al. 2014b). Differentiation of 3T3-L1 cells with PA or OA supplemented medium increased cyclophilin A in cell media and this is considered an indicator of early necrosis (Christofferson and Yuan 2010). Number of 3T3-L1 cells exposed to PA or OA during differentiation did, however, not decline (Krautbauer et al. 2014b). Hence, induction of cell death seems not to contribute to higher Tgfb in 3T3-L1 cell media. 3T3-L1 cells differentiated in the presence of TNF and IL-6 had lower levels of cellular Tgfb and higher Tgfb in cell media indicating that depletion of cellular Tgfb contributes to the elevation in cell media.

Many different cell types such as macrophages and endothelial cells can secrete Tgfb (Frangogiannis 2020). Activation of latent Tgfb is tightly controlled and Tgfb signaling is highly complex and not completely understood (Frangogiannis 2020). Though increased expression of Tgfb mRNA and protein (Fain et al. 2005; Kumar et al. 2019) in obese fat tissues suggested higher activity of this cytokine, this needs to be confirmed by functional assays. Non-coordinate regulation of Tgfb and its target gene Ccn2 shows that adipose tissue and adipocytes produced Tgfb are not biologically active. Tgfb signaling is tightly regulated, and different antagonists such as Bambi (Sekiya et al. 2004) may have a role herein.

Leptin is a very well described adipokine whose circulating levels are increased in the obese. Notably, leptin is a pro-fibrotic protein and induces cardiac and liver fibrosis (Martinez-Martinez et al. 2014; Wang et al. 2009). Leptin increased Tgfb and Ccn2 release of Kupffer cells, which subsequently activate hepatic stellate cells (Wang et al. 2009). Current findings suggest that leptin also enhances adipocyte Tgfb production. However, Ccn2 was not induced in parallel.

Excess free fatty acids cause insulin resistance and inflammation. Persistent inflammation induces repair mechanisms and tissue fibrosis (Capurso and Capurso 2012; Krautbauer et al. 2014c; Nath et al. 2015). PA and OA were already shown to increase TGFb in cell media of primary human hepatocytes (Wanninger et al. 2011) and likewise elevated its levels in adipocyte media. Again, cellular Ccn2 protein did not change upon incubation of adipocytes with these fatty acids.

LPS translocation from the gut is higher in obesity and contributes to metabolic diseases (Troseid et al. 2013). LPS was shown to induce TGFb activity in cell media of dendritic cells and to enhance TGFb induced Ccn2 production of fibroblasts (Burke et al. 2010; Fenton et al. 2017). LPS also upregulated Ccn2 protein in epithelial cells (Nishioka et al. 2010). Cellular Ccn2 protein did, however, not increase in 3T3-L1 cells treated with LPS. Cell media Ccn2 protein was quite low and could not be measured by ELISA.

Bambi antagonizes Tgfb activity (Sekiya et al. 2004) and its regulation in 3T3-L1 cells was also studied. Bambi expression did not change in 3T3-L1 cells by the addition of LPS, leptin, or fatty acids to cell media. LPS lowered BAMBI mRNA and protein in hepatic stellate cells (Seki et al. 2007) showing that the effect of LPS on BAMBI expression is cell-type specific.

This study has limitations. Controls for ob/ob mice were WT mice and not heterozygous animals. Heterozygous mice in comparison to WT controls have less leptin, higher fat mass (Chung et al. 1998) and increased fasting glucose (Flatt and Bailey 1981). Additional analysis of heterozygous mice may help to further define obesity related and metabolic disease associated changes in fat tissues. Further limitation of our study is that Tgfb and Col1a1 protein could not be detected by the experimental protocol used.

Translation of results from murine studies to human obesity is the aim of research. Current results indicate that mRNA and protein levels of, e.g. Col6a1 and Acta2 are not correlated suggesting that protein analysis is essential. Discordant results regarding *COL6A3* mRNA in human obesity, with higher and lower levels in subcutaneous fat, have been reported. Body mass index differs by about 10 kg/m^2^ between these two obese cohorts (McCulloch et al. 2015; Dankel et al. 2020), and expression of fibrosis-related genes and proteins may change with the grade of obesity as was observed for HFD and ob/ob mice herein. To sum up, current study shows that the expression and obesity related regulation of fibrosis-associated genes varies between the different white fat depots and brown fat. Importantly, expression of *Acta2* mRNA in ob/ob mice was not in concordance with the regulation of the respective protein. Upregulation of *Tgfb* was detected in all white fat depots of the overweight/obese mice but this was not consistently accompanied by an increase of Tgfb target genes suggesting that Tgfb activity differs between the fat depots.

### Supplementary Information

Below is the link to the electronic supplementary material.

Supplementary file1 (DOCX 1924 kb)

## Data Availability

All data generated are included in this article. Original data are available from the corresponding author on request.
