# Supplementary material for: Adipose tissue depot specific expression and regulation of fibrosis-related genes and proteins in experimental obesity
Source: Mamm Genome. 2023 Oct 26;35(1):13–30. doi: 10.1007/s00335-023-10022-3 (PMC10884164; doi:10.1007/s00335-023-10022-3)
Supplement: Supplementary file 1 — Supplementary file1 (DOCX 1924 kb) [file 335_2023_10022_MOESM1_ESM.docx]

Adipose tissue depot specific expression and regulation of fibrosis-related genes and proteins in experimental obesity

Kristina Eisinger^1^, Philipp Girke^2^, Christa Buechler^1,*^, Sabrina Krautbauer^1^

^1^ Department of Internal Medicine I, Regensburg University Hospital, 93053 Regensburg, Germany

^2^ Department of Genetics, University of Regensburg, 93040 Regensburg, Germany

* Address correspondence to: [christa.buechler@klinik.uni-regensburg.de](mailto:christa.buechler@klinik.uni-regensburg.de)


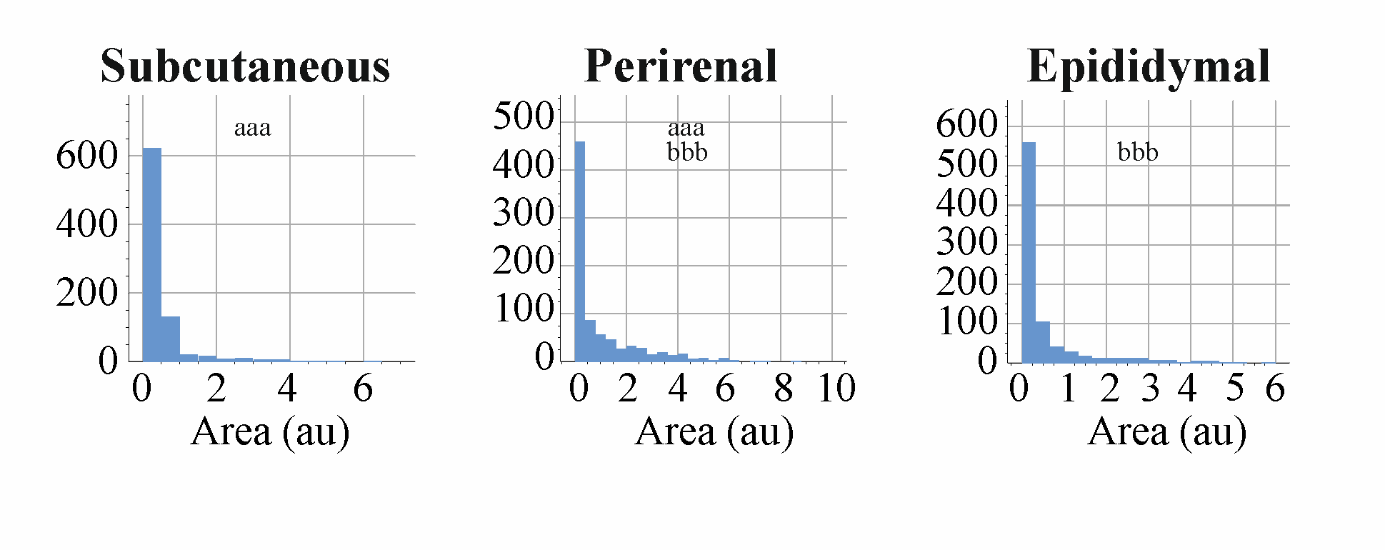


**Supporting Fig. 1** Adipocyte area of different fat depots of mice fed a standard chow. Identical letters in the figures indicate significantly different areas between these two tissues; aaa, p < 0.001; bbb, p < 0.001.


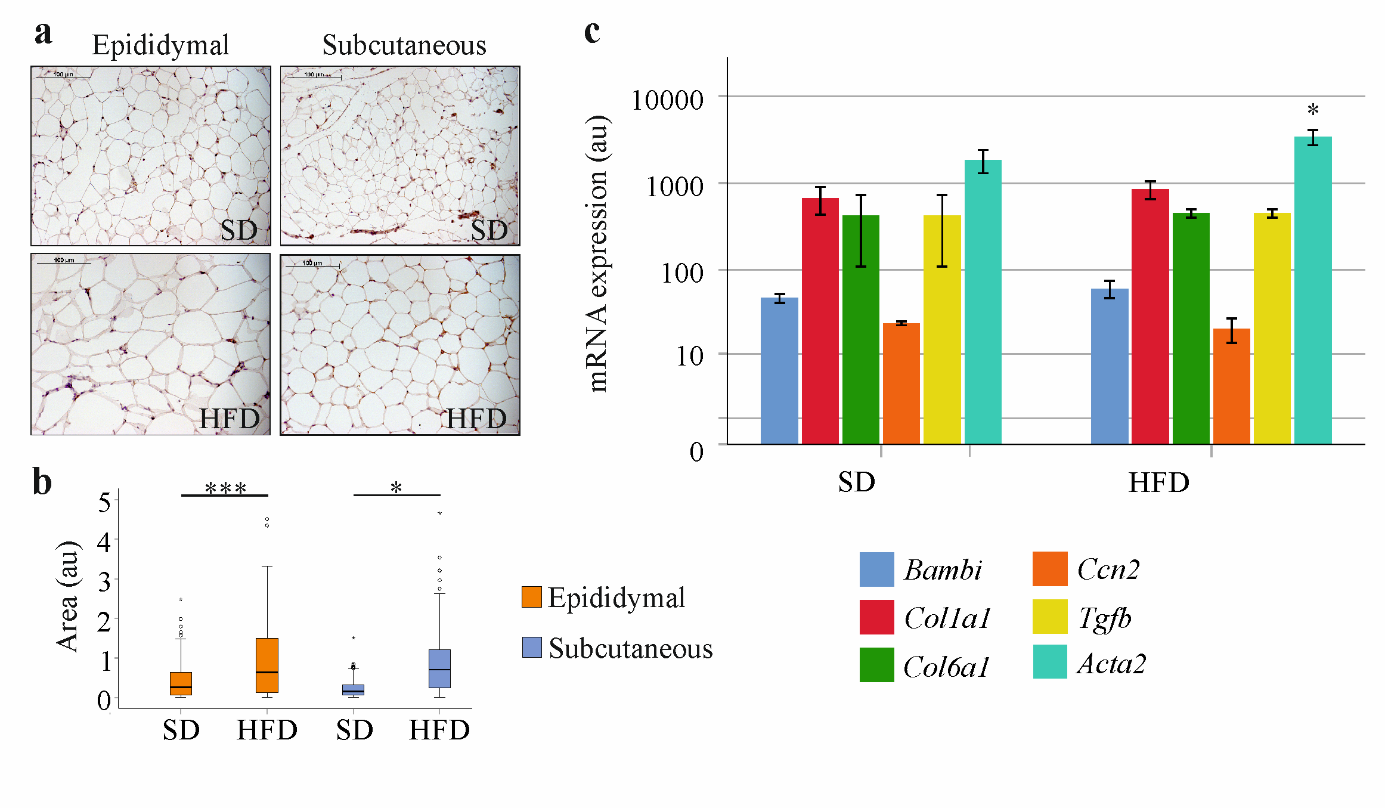


**Supporting Fig. 2** Adipocyte area of mice fed a standard diet (SD) or high fat diet (HFD) and expression of genes with a role in fibrosis in subcutaneous fat of mice. **a** H&E stained fat tissues of 21 week old mice fed a SD or HFD for the last 14 weeks. **b** Adipocyte areas of epididymal and subcutaneous fat (n = 5 per group). **c** mRNA expression of subcutaneous fat as determined by GeneChip analysis. Subcutaneous fat of 28 week old male C57BL/6 mice fed a HFD or SD for the last 14 weeks was used (n = 5 per group). * p < 0.05,*** p < 0.001.


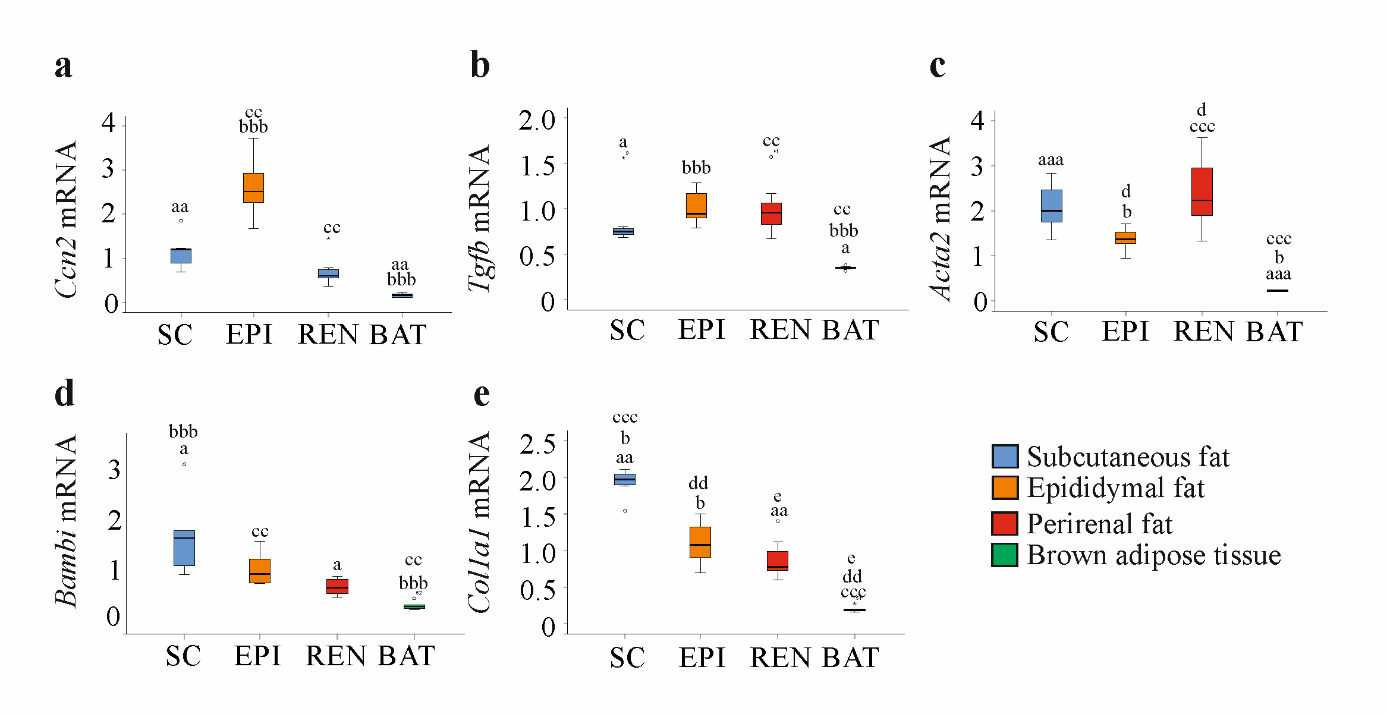
 **Supporting Fig. 3** Expression of *Ccn2*, *Tgfb*, *Acta2*, *Bambi* and *Col1a1* mRNA in subcutaneous (SC), epididymal (EPI), perirenal (REN) and brown adipose tissue (BAT) of 21 week old mice fed a high fat diet for the last 14 weeks. Expression of **a** *Ccn2*. **b** *Tgfb*. **c** *Acta2*. **d** *Bambi* and **e** *Col1a1* in fat tissues. (n = 7 per group). Identical letters in the figures indicate significantly different expression between these tissues. p < 0.05 (one letter), p < 0.01 (two letters), p < 0.001 (three letters).





**Supporting Fig. 4** Correlation of *Tgfb* and *Col1a1* mRNA with *Adgre* (coding for the c**ell surface glycoprotein F4/80)** mRNA and blood glucose in mice fed either a standard diet or a high fat diet. Correlation of **a** perirenal (ren) *Tgfb* and **b** ren *Col1a1* mRNA with blood glucose. **c** Correlation of subcutaneous (sc) *Tgfb* mRNA with sc *Adgre* mRNA. **d** Correlation of ren *Tgfb* mRNA with ren *Adgre* mRNA (n = 7 per group). * p < 0.05, *** p < 0.001.


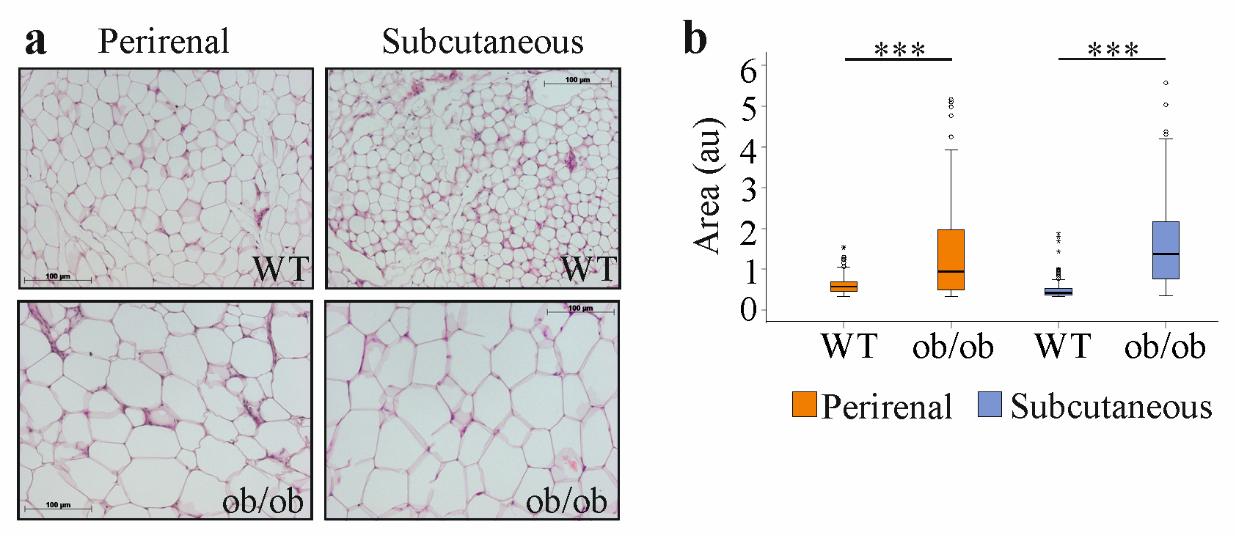


**Supporting Fig. 5** Adipocyte area of wild type (WT) and ob/ob mice. **a** H&E stained fat tissues of WT and ob/ob mice. **b** Adipocyte areas of the white fat depot (n = 5 per group). *** p < 0.001.


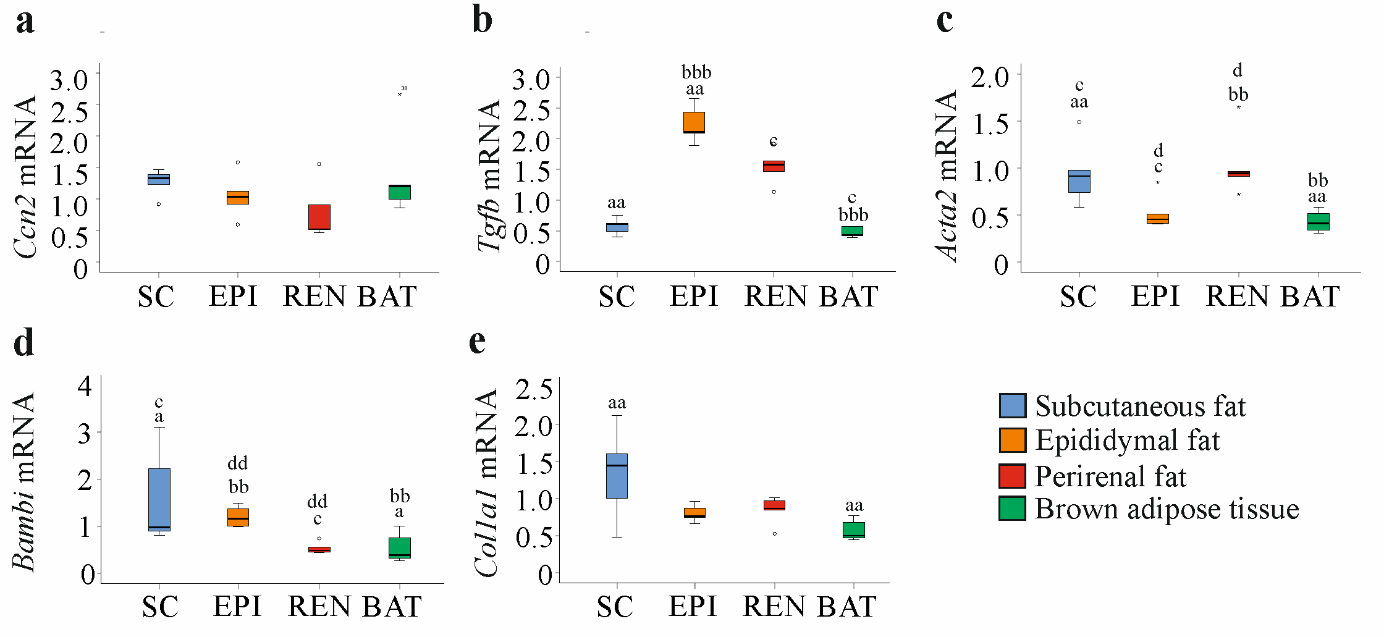


**Supporting Fig. 6** Expression of *Ccn2*, *Tgfb*, *Acta2*, *Bambi* and *Col1a1* mRNA in subcutaneous (SC), epididymal (EPI), perirenal (REN) and brown adipose tissue (BAT) of ob/ob mice. Expression of **a** *Ccn2*. **b** *Tgfb*. **c** *Acta2*. **d** *Bambi* and **e** *Col1a1* in fat tissues. Identical letters in the figures indicate significantly different expression between these tissues (n = 5 per group). p < 0.05 (one letter), p < 0.01 (two letters), p < 0.001 (three letters).

**
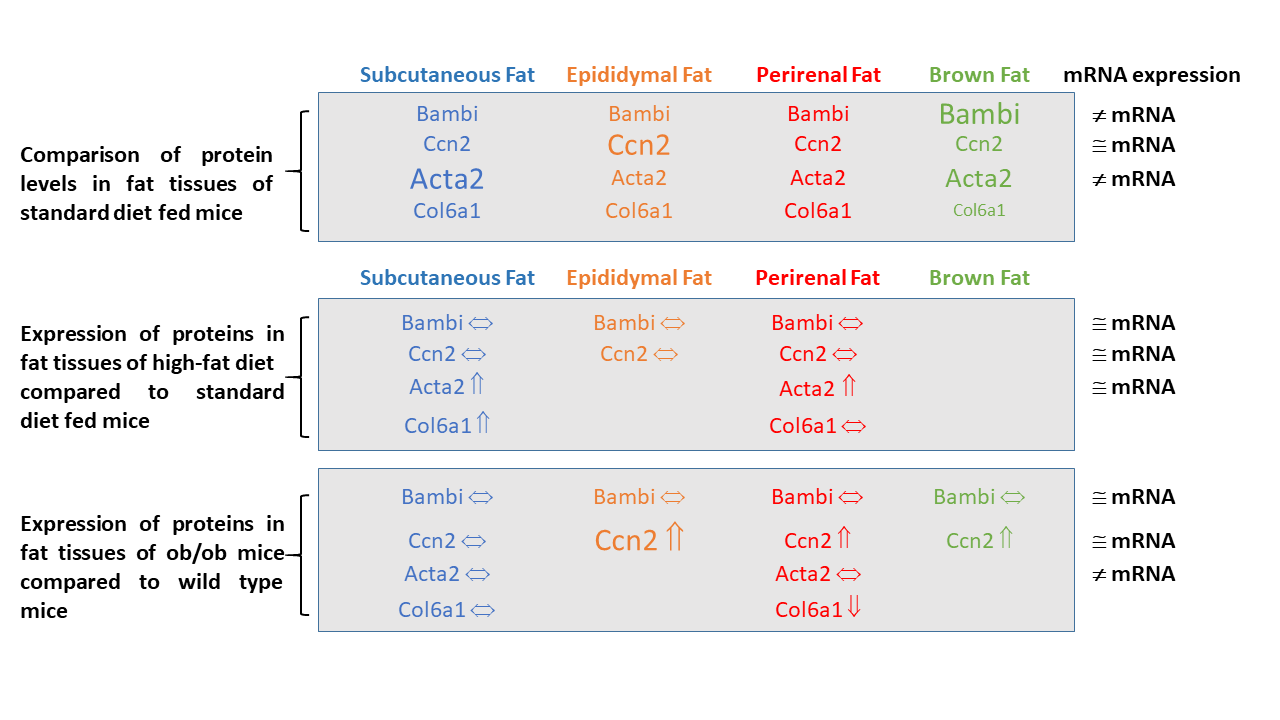
**

**Supporting Fig. 7** Summary of protein expression in the different fat depots and changes in obesity.

Panel 1 compares the protein expression in the different fat depots of standard diet fed mice. Larger letters indicate higher levels. Panel 2 compares the protein levels of mice fed a high fat diet and the respective controls. Panel 3 compares the protein levels of ob/ob mice and the respective controls. ⇔

no change in expression, ⇑ higher expression in high fat diet / ob/ob, ⇓ lower expression in ob/ob. The last column indicates whether protein expression correlates more or less with mRNA data ( ≅) or does not correlate with mRNA expression (≠).
